# Unusual Presentation and Surgical Treatment of a Phosphaturic Mesenchymal Tumor in a Knee

**DOI:** 10.3389/fsurg.2022.746623

**Published:** 2022-05-25

**Authors:** Juan Sun, Xi Zhou, Weibo Xia, Huanwen Wu, Shuzhong Liu, Huizhen Wang, Yong Liu

**Affiliations:** ^1^ Department of General Surgery, Peking Union Medical College Hospital, Chinese Academy of Medical Sciences and Peking Union Medical College, Beijing, China; ^2^ Department of Orthopedic Surgery, Peking Union Medical College Hospital, Chinese Academy of Medical Sciences and Peking Union Medical College, Beijing, China; ^3^ Department of Endocrinology, Peking Union Medical College Hospital, Chinese Academy of Medical Sciences and Peking Union Medical College, Beijing, China; ^4^ Department of Pathology, Peking Union Medical College Hospital, Chinese Academy of Medical Sciences and Peking Union Medical College, Beijing, China; ^5^ Department of Operating Room, Peking Union Medical College Hospital, Chinese Academy of Medical Sciences and Peking Union Medical College, Beijing, China

**Keywords:** tumour-induced osteomalacia, knee joint, surgery, phosphaturic mesenchymal tumors, polymethylmethacrylate (PMMA)

## Abstract

A 30-year-old woman presented to our hospital with an 11-year history of gradually enlarging masses around the left knee and 2-year history of progressively worsening bone pain. Tumor-induced osteomalacia (TIO), a rare paraneoplastic syndrome caused by phosphaturic mesenchymal tumors (PMTs) was suspected, but the postoperative pathology of her two operations was both reported as tenosynovial giant cell tumor (TGCT), making its diagnosis confusing. The possibility of hypophosphatemia, insufficient blood supply, innervation of the left lower limbs, as well as the unclear pathology, make it unreasonable to perform tumor-type knee prosthesis replacement directly. Finally, we placed static polymethylmethacrylate (PMMA) spacer at first, then when the concentration of blood phosphorus level rose to the normal range, the pathology was confirmed to be TIO, the blood supply and innervation was satisfying, tumor-type knee prosthesis replacement was performed. She was discharged post operative day 15 after the prothesis implantation without incident. One and a half years after her surgery, the concentration of blood phosphorus was still in the normal range, the symptom of systemic bone pain had improved significantly, the prosthesis was still in a good position and no recurrence was caught.

## Introduction

Tumor-induced osteomalacia (TIO) is a rare paraneoplastic syndrome caused by the overproduction of fibroblast growth factor 23 (FGF23) secreted by phosphaturic mesenchymal tumors (PMTs). In TIO, chronic hypophosphatemia induced by FGF23 leads to a suboptimal supply of phosphate to bone and a subsequently reduced rate of osteoid mineralization, resulting in osteomalacia in adults and rickets in children (1). Histologically, TIO is a kind of PMT with the characteristics of high vascularization and proliferation of spindle- and stellate-shaped cells. These cells produce a matrix similar to the original cartilage or cartilage-like matrix and often contain numerous osteoclast-like giant cells and occasionally ossification (2). And the spindled bland neoplastic cells of PMT can produce smudgy basophilic matrix material which often calcifies to form a distinctive grungy calcification (3). The biggest difficulty of its treatment is to find the responsible lesions since they are usually well hidden. But once the lesion is located, tumor resection is the established, definitive and most effective therapeutic method.

Here we reported a rare case about a young lady with a diffuse tumor around the knee joint initially operated on and diagnosed as a chondroma, subsequently reoperated and diagnosed as a tenosynovial giant cell tumor (TGCT). The posterior knee neurovascular bundle was closely involved with the tumor and there was pre-opeartive concern that they could be compromised, severly limiting our ability to perform successful limb-salvage. Ultimately, resolution of the TIO after aggressive surgical resection made it more likely the tumor was a PMT.

The diagnosis and proper treatment to avoid hypophosphatemia, insufficient blood supply and innervation of the left lower limbs were the challenges in our case. And to our knowledge, this is the first case ever has been reported in terms of this modus operandi for the aggressive TIO around the knee joint.

## Case Report

A 30-year-old woman presented to our hospital in July 2020, with an 11-year history of gradually enlarging masses around the left knee joint and 2-year history of progressively worsening bone pain. In 2009, when she was 19 years old, a mass of 2 × 2 cm with no erythema, swelling or pain was found on the lateral side of her left knee. When the mass eventually grew to 4 × 5 cm, the patient noticed the mass due to accompanying swelling and pain with ambulation. In March 2011, she went to see the doctor at a local hospital, but the concentration of blood phosphorus was unrecorded at that time. Tumor resection around the left knee joint was performed, and the postoperative pathology was consistent with that of chondroma. Since then, the discomfort of the left knee was partially relieved after this operation. However, from 2011 to 2016, the tumor relapsed and gradually spread around the knee joint, which limited her walking. Then she underwent tumor resection again in 2017 and the pathology was diffuse TGCT. After that, the pain and other symptoms were completely relieved, but the magnetic resonance imaging (MRI) taken every six months still showed diffuse abnormal signals around the left knee joint.

In July 2018, the patient went to a larger hospital with the complaint of the pain in lower back and left knee joint when moving. The MRI revealed multiple cystic lesions in the left popliteal fossa soft tissue and the first record of the concentration of blood phosphorus was 0.47 mmol/L at that time (normal range: 0.81–1.45 mmol/L). Tumor resection was done again and postoperative pathology was still TGCT. But unfortunately, her back pain was gradually aggravated following this operation. In September 2019, the patient came to our hospital presenting with progressively worsening bone pain and inability to walk independently. The patient did not have any history of smoking, alcohol or drug use.

**Figure 1:**
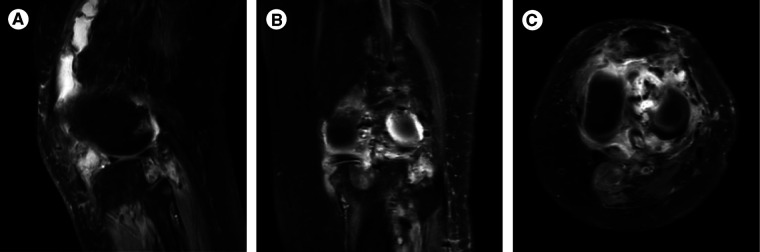
MRI of the left knee joint: multiple abnormal nodules in the articular cavity, posterior tibial space, and popliteal space, which are presented as isosignals on T1WI and mixed signals on T2WI. (**A**) Median sagittal section. (**B**) Coronal section. (**C**) Transverse section.

**Figure 2:**
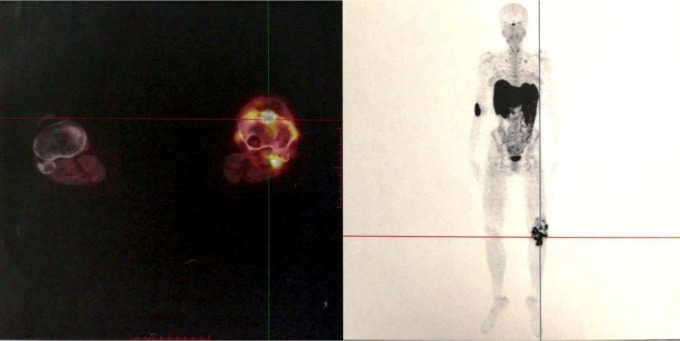
^68^Ga-DOTATATE-PET/CT: multiple lesions with high levels of somatostatin receptor in the soft tissue around the left knee joint and the proximal left tibia.

On examination, she had normal vital signs and limited motion of the left knee. She could ambulate 5 meters. Muscle strength was 4/5 for both legs. The concentration of blood phosphorus was 0.57 mmol/L, alkaline phosphatase (ALP) was 240 U/L (normal range: 35–100 U/L), 1,25(OH)_2_D was 1,71 pg/mL (normal range: 19.6–54.3 pg/mL) and total-25(OH)D was 11.5 ng/mL (<20 ng/mg was defined as lacking), and the further testing with phosphorus clearance and load tests verified her loss of phosphorus was renal in origin. Magnetic Resonance Imaging (MRI) demonstrated multiple abnormal nodules in the articular cavity, posterior tibial space, and popliteal space (Figure 1), which all had high levels of somatostatin receptor-based on ^68^Ga-DOTATATE-PET/CT scans (Figure 2).

Based on the persistent hypophosphatemia together with findings of MRI and ^68^Ga-DOTATATE-PET/CT, tumor-induced osteomalacia (TIO) around the knee joint was suspected. The family history of inherited disorders of phosphate metabolism was excluded. But the postoperative pathology results of the last two operations were both reported to be TGCT, which confused us most.

**Figure 3:**
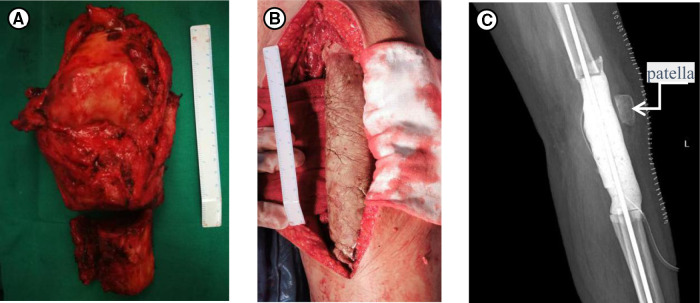
(**A**) The specimens removed during the first operation. (**B**) Intraoperative photograph of the temporary PMMA intervening device of left knee joint. (**C**) Postoperative X-rays taken after the first surgery.

**Figure 4:**
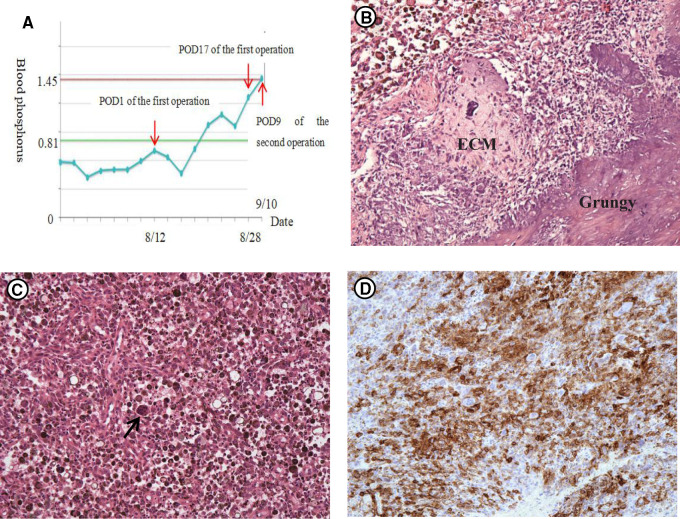
(**A**) The change of the concentration of blood phosphorus (mmol/L). The first operation was performed on August 11 and her blood phosphorus was normal since post-operation of day 6 (POD6). The second operation was performed on September 1st and the blood phosphorus was still normal on POD9. (**B,C**) HE staining (×100): Tenosynovial giant cell tumor, diffuse type. (**B**) Characterized “grungy” and chondroid extracellular matrix (ECM). (**C**) Tumors are composed of an admixture of histiocyte-like cells, larger epithelioid cells with vesicular nuclei, and osteoclast-like giant cells. The cytoplasm of these cells contains abundant haemosiderin qranules. (**D**) Immunohistochemical staining of SSTR2 (×100) demonstrating that tumor cells express somatostatin receptors.

**Figure 5:**
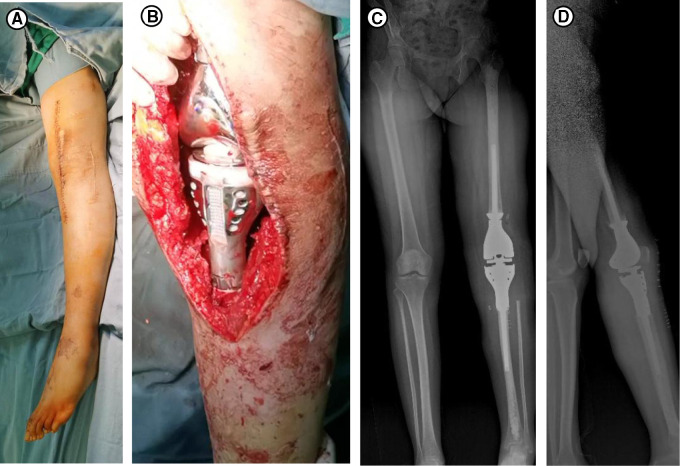
(**A**) Disinfection for the second operation. (**B**) Prosthesis of the left knee joint. (**C,D**) X-rays taken 2 weeks after the second surgery, (**C**) is anteroposterior and (**D**) is lateral.

In terms of her treatment, she had taken phosphate supplements and 1,25(OH)_2_D for nearly one year prior to her arrival at our hospital, but the hypophosphatemia was still not cured. Therefore, oral phosphorus for her whole life seemed unlikely to work. The curative effect of performing tumor-type knee prosthesis replacement directly remained unclear, because the postoperative blood phosphorus concentration, the existence of blood supply and innervation of the left lower limbs were unforeseeable. Furthermore, the pathology was not clear yet. If none of the choices above worked, amputation would be inevitable, and the prosthesis worth tens of thousands of dollars would have been used in vain. Importantly, the patient could not accept amputation and that limb salvage was feasible since TIO was a benign tumor in some degree. Therefore, we developed a rigorous, individualized treatment plan for her. Firstly, we removed the tumors around the left knee, including the knee joint. Then, a temporary static PMMA spacer was placed (Figure 3). The patient returned to the ward after surgery and was monitored for changes in the concentration of blood phosphorus. When the concentration of blood phosphorus increased and stabilized at normal levels and the postoperative pathology confirmed that it was a phosphaturic mesenchymal tumor (PMT) (Figure 4). We therefore performed left knee joint tumor-type prosthesis replacement three weeks after the first operation (Figure 5).

## Discussion

An estimated 500 cases of TIO with typical small tumor producing FGF23 and causing hypophosphatemia in adults have been reported (4). Patients with TIO always complain of progressive musculoskeletal pain and muscle weakness with fractures caused by bone insufficiency. Fractures and skeletal deformities can occur in severe cases, and as can loss of height as a result of multiple vertebral fractures. PMTs which are responsible for causing the disease can be very difficult, or even impossible to find, as they may be located within soft tissue or bone anywhere in the body. According to the reports, PMTs were mostly located in the lower limbs (53.9%), head (29.2%), upper limbs (6.7%), chest and abdomen (5.6%), or pelvis (4.5%) (5–10).

Due to the nonspecific symptoms and difficulty in locating tumors, the diagnosis of TIO is often elusive. And according to statistics, latency to diagnosis is 4 years by median and can take up to 22 years (4). When TIO is suspected, and if the patient’s condition permits, phosphate, creatinine, and calcium levels in serum and urine should be measured, as well as serum PTH, 1,25(OH)_2_D, FGF23, total or alkaline phosphatase, proteins (by electrophoresis). But none of these biomarkers are specific. PMTs are notoriously difficult to locate. The first step in locating the responsible foci is to collect a complete medical history and conduct a thorough physical examination. Next, imaging examinations should be carried out: functional imaging is the initial step, followed by anatomical imaging (4). More recently, somatostatin receptor (SSTR) analogues conjugated to positron-emitting isotopes such as ^68^Ga have become available and can be used to locate the PMTs. MRI may be successful in some cases but usually considered to be neither sensitive nor specific for TIO (1).

The main reason that prevented us from diagnosing TIO in this case was the postoperative pathology results of the last two operations: both were reported as TGCTs. When we repeatedly combed the patient’s medical history and checked the postoperative pathological sections, we found the mass was a PMT which expressed somatostatin receptor (Figures 4B,D) but presented as a TGCT (Figure 4C). However, 4 years ago, when the postoperative pathology was TGCT, there was no bone pain and hypophosphatemia. We suspect that at that point, it was indeed TGCT. However, the TGCT observed 2 years ago may have not actually been the TGCT, but may have mutated into somatostatin receptor-expressing PMT, given that bone pain and hypophosphatemia were also present at that time.

Surgery is the established, definitive and most effective treatment of TIO. Some severe cases may need two or more surgeries to remove the entire tumor and/or replace the damaged tissue, for example with prosthesis replacement, as in our report. The innovation in our treatment plan of TIO was to place a static polymethylmethacrylate (PMMA) spacer in the tumor patient to wait until the conditions were suitable for a tumor-type knee prosthesis, which has not previously been done. Once the tumor causing TIO has been located and successfully removed, the typical biochemical abnormalities of this condition are reversed and patients’ symptoms start to improve within days or weeks after surgery, but the bone healing may take up to a year or more (4). When the tumor cannot be located or completely resected, other medical treatment is necessary which mainly involves the use of phosphate supplements and 1,25(OH)_2_D. When surgery with wide margins cannot be performed, adjuvant radiotherapy is recommended to avoid recurrence, but it is still controversial and has limited use (1). Other treatments also have been reported, such as calcium-sensitive receptor agonist cinacalcet to induce hypoparathyroidism (11), octreotide receptor (12), anti-FGF23 antibody (13), and radiofrequency ablation (14), etc. However, most of these are from case reports and have mainly been used in cases where TIO is highly suspected but the tumor has not been located.

The patient in our report was discharged on the 15^th^ day after the second operation and the wound healed well at that time. The tumors around her knee were completely removed and her pain was relieved. Furthermore, the patient was highly satisfied that limb salvage could be performed. One and a half years after her surgery, the concentration of blood phosphorus was still in the normal range and the symptom of systemic bone pain had improved significantly, based on a visual analogue scale (VAS) score that dropped from 9 to 1. She can now walk for at least 50 meters by herself without the help of canes. What’s more, the latest full-length X-ray of lower limbs showed that her knee joint tumor-type prosthesis was in a good position and there was no recurrence of the tumor.

## Data Availability

The original contributions presented in the study are included in the article/Supplementary Material, further inquiries can be directed to the corresponding author/s.
